# SOX17 overexpression sensitizes chemoradiation response in esophageal cancer by transcriptional down-regulation of DNA repair and damage response genes

**DOI:** 10.1186/s12929-019-0510-4

**Published:** 2019-02-18

**Authors:** I-Ying Kuo, Yu-Lin Huang, Chien-Yu Lin, Chien-Hsun Lin, Wei-Lun Chang, Wu-Wei Lai, Yi-Ching Wang

**Affiliations:** 1 Department of Pharmacology, College of Medicine, National Cheng Kung University, No.1, University Road, Tainan, 70101 Taiwan; 2 Institute of Basic Medical Sciences, College of Medicine, National Cheng Kung University, No.1, University Road, Tainan, 70101 Taiwan; 3 Department of Internal Medicine, National Cheng Kung University Hospital, College of Medicine, National Cheng Kung University, No.138, Sheng Li Road, Tainan, 704 Taiwan; 4 Department of Surgery, National Cheng Kung University Hospital, College of Medicine, National Cheng Kung University, No.138, Sheng Li Road, Tainan, 704 Taiwan

**Keywords:** SOX17, Transcription regulation, Resistance, DNA repair, Concurrent chemoradiation therapy, Esophageal cancer

## Abstract

**Background:**

Prognosis of esophageal squamous cell carcinoma (ESCC) patients is poor and the concurrent chemoradiation therapy (CCRT) provided to ESCC patients often failed due to resistance. Therefore, development of biomarkers for predicting CCRT response is immensely important. In this study, we evaluated the predicting value of SRY (sex determining region Y)-box 17 (SOX17) protein during CCRT and its dysregulation of transcriptional targets in CCRT resistance in ESCC.

**Methods:**

Pyrosequencing methylation, RT-qPCR and immunohistochemistry assays were performed to examine the DNA methylation, mRNA expression and protein expression levels of SOX17 in endoscopic biopsy from a total of 70 ESCC patients received CCRT. Cell proliferation, clonogenic survival and xenograft growth were used to confirm the sensitization of ESCC cell line KYSE510 in response to cisplatin, radiation or CCRT treatment by SOX17 overexpression in vitro and in vivo. Luciferase activity, RT-qPCR and ChIP-qPCR assays were conducted to examine transcription regulation of SOX17 in KYSE510 parental, KYSE510 radio-resistant cells and their derived xenografts.

**Results:**

High DNA methylation coincided with low mRNA and protein expression levels of SOX17 in pre-treatment endoscopic biopsy from ESCC patients with poor CCRT response. SOX17 protein expression exhibited a good prediction performance in discriminating poor CCRT responders from good responder. Overexpression of SOX17 sensitized KYSE510 radio-resistant cells to cisplatin, radiation or CCRT treatment in cell and xenograft models. Importantly, SOX17 transcriptionally down-regulated DNA repair and damage response-related genes including *BRCA1*, *BRCA2*, *RAD51*, *KU80 DNAPK, p21, SIRT1, NFAT5* and *REV3L* in KYSE510 radio-resistant cells to achieve the sensitization effect to anti-cancer treatment. Low expression of BRCA1, DNAPK, p21, RAD51 and SIRT1 was confirmed in SOX17 sensitized xenograft tissues derived from radio-resistant ESCC cells.

**Conclusions:**

Our study reveals a novel mechanism by which SOX17 transcriptionally inactivates DNA repair and damage response-related genes to sensitize ESCC cell or xenograft to CCRT treatment. In addition, we establish a proof-of-concept CCRT prediction biomarker using SOX17 immunohistochemical staining in pre-treatment endoscopic biopsies to identify ESCC patients who are at high risk of CCRT failure and need intensive care.

**Electronic supplementary material:**

The online version of this article (10.1186/s12929-019-0510-4) contains supplementary material, which is available to authorized users.

## Background

Esophageal cancer is one of the most aggressive cancers and the seventh leading cause of cancer death worldwide [1]. Squamous cell carcinoma is the major histological type of esophageal cancer in endemic areas, such as Eastern Asia [2, 3]. More than 75% of esophageal squamous cell carcinomas (ESCCs) are diagnosed at advanced stages, for which concurrent chemoradiotherapy (CCRT) has been recommended as the first-line treatment, either with or without following esophagectomy [4–6]. In such patients, treatment response to CCRT is the major determining factor of patient outcome. However, recent studies suggest that ESCC patients have diverse responses to CCRT. Not all patients respond, and many develop acquired resistance to therapy [7–10]. Therefore, development of sensitive and specific molecular biomarkers for predicting CCRT response is urgently needed. In addition, evaluation of the mechanistic effects of resistance to CCRT in ESCC is important.

Accumulating evidence has revealed that silenced tumor suppressor-like genes could be potential prognostic biomarkers in ESCC, such as *APC* [11], *CDO1* [12], *CLDN4* [13], *E-caherin* [14], *FHIT* [15, 16], *p16* [17, 18], *RARβ2* [18, 19], *RASSF1A* [16], *RASSF5A* [20], *RUNX3* [21], *SFRP1* [22], *SLIT2* [23], *Tachykinin-1* [24], and *TIP30* [25] genes. We and others have previously reported the dysregulated tumor suppressive function of SOX17 [SRY (sex determining region of Y chromosome)-box 17] transcription factor in ESCC [26, 27]. Overexpression of SOX17 suppresses cell colony formation in soft agar and migration/invasion ability in ESCC cell model. In addition, SOX17 inhibits tumor growth and metastasis in ESCC xenograft animal model. Notably, promoter hypermethylation of *SOX17* gene leading to silence of SOX17 protein can be found in tumor of ~ 50% ESCC patients analyzed [26]. These results indicated that *SOX17* acts as tumor suppressor gene and plays an important role in ESCC tumorigenesis processes. However, the role of SOX17 in anti-cancer therapy response remains unclear.

Up to date, most of the studies on biomarkers of response and resistance to anti-cancer treatment have focused on either chemotherapy or radiotherapy [10] and the underlying mechanisms of dysregulated biomarkers remain unclear. Our previous study established the six-CpG panel of DNA methylation biomarkers including *IFNGR2*, *KCNK4*, *NOTCH4*, *NPY*, *PAX6* and *SOX17* for CCRT response prediction in pre-treatment endoscopic biopsies from ESCC patients with known CCRT responses during follow-up [28]. In the current study, we have shown that low SOX17 protein expression, which could be analyzed by immunohistochemisty in pre-treatment endoscopic biopsies, is associated with poor CCRT response of ESCC patients. Re-expression of SOX17 was confirmed to sensitize radio-resistant ESCC cells to CCRT treatment in cell and xenograft models. Mechanistically, SOX17 transcriptionally inactivated DNA repair and damage response genes and contributed to the sensitization effects to chemoradiation.

## Methods

### Patients and endoscopic tissue samples

A total of 70 ESCC patients who received concurrent chemoradiotherapy (CCRT) as their initial treatment were recruited consecutively from endoscopic room of National Cheng Kung University Hospital since March 2009 to January 2015. Appropriate institutional review board permission and informed consent from the patients were obtained. The CCRT protocol included radiotherapy for esophageal tumor and regional lymph nodes with 1.8 Gy (Gy) per day and 5 days per week and either one of the two standard chemotherapy regimens given concomitantly as described in our previous publication [28]. The treatment responses were evaluated by endoscopic ultrasonography (EUS) and computed tomographic (CT) scans from chest to pelvic region, and PET-CT scan when necessary, after completion of 36 Gy radiotherapy. Patients whose radiotherapy doses did not achieve 50 Gy or did not complete chemotherapy course due to toxicity were excluded. The CCRT response criteria, which define patients with post-treatment esophageal wall thickness < 8 mm as good responder, have been validated in our previous studies [28, 29]. The patients’ pre-treatment endoscopic biopsy samples were analyzed for DNA methylation and mRNA expression and the embedded paraffin blocks were examined for protein expression.

### Cell lines and culture conditions

ESCC cell line KYSE510 was purchased from the DSMZ-German Collection of Microorganisms and Cell Cultures (Braunschweig, Germany), where they were characterized by DNA-fingerprinting and isozyme detection. Cells were cultured in RPMI1640 medium (Gibco, Invitrogen, Carlsbad, CA, USA). The KYSE510 radio-resistant cell line (KYSE510-R) was generously provided by Dr. Fong-Chia Lin, the Division of Radiation Oncology, National Cheng Kung University Hospital. The KYSE510-R cell line was developed by exposing the parental KYSE510 cells to radiation dose of 5 Gy per treatment. After each treatment, cells were allowed to recover and the next treatment was given when cells reached 50% confluency until a total radiation dose of 70 Gy. All media were supplemented with 10% Fetal Bovine Serum (Gibco) and 1% penicillin/streptomycin (Gibco). All cells were incubated at 37 °C in a humidified incubator containing 5% CO_2_ in air.

### Expression vectors, promoter constructs, siRNA and transfection

The plasmids used in the study are listed in Additional file 1: Table S1. The SOX17 expression construct was kindly provided by Dr. Stephen B. Baylin at Division of Cancer Biology, Sidney Kimmel Comprehensive Cancer Center, Johns Hopkins University. The entire coding region of *SOX17* cDNA was subcloned in frame into the pcDNA3.1/V5-His B vector (Invitrogen). To generate cells that stably expressed SOX17, SOX17 expression vector (KYSE510-SOX17) and empty vector (KYSE510-EV) were transfected into the KYSE510 cell line with Turbofect (Thermo Scientific, Waltham, MA, USA) according to the manufacturer’s protocol. Single stable clone either KYSE510-SOX17 or KYSE510-EV was selected using G418 (1000 μg/ml, Gibco). To examine the CCRT-sensitization function, SOX17 expression vector (KYSE510-R-SOX17) was transfected into the KYSE510 radio-resistant cell line using Turbofec. Empty vector–transfected cells were used as control (KYSE510-R-EV). SOX17 siRNA pools (SASI_Hs01_00021141, SASI_Hs01_00021142 and SASI_Hs02_00355717) were purchased from Sigma-Aldrich (St. Louis, MO, USA).

To construct the *p21 promoter*-Luciferase reporter plasmid, the DNA fragment corresponding to residues − 2276~ + 61 containing 24 SOX17 binding sites (predicted by PROMO) was PCR amplified. The PCR product was cloned into the pGL3 basic (Promega, Madison, WI, USA) to obtain pGL3-*p21*-Luc promoter plasmid. The procedures generating pGL3-*NFAT5*-Luc promoter plasmid are as previously described [26]. KYE510-R cells (4 × 10^4^ per well) co-transfected with 2 μg/well of SOX17 expression vector or empty vector, 2 μg/well of the pGL3-luc vector, pGL3-*p21*-Luc or pGL3-*NFAT5*-Luc and 1 μg of Renilla plasmid (as an internal control) were subjected for the dual luciferase reporter assay system (Promega).

### Pyrosequencing methylation assay

To quantify the methylated cytosine in the six analyzed CpG sites of the promoter region of *SOX17* gene in tumor and corresponding normal samples, bisulfite-converted DNA was analyzed by a pyrosequencing system (PyroMark Q24, Qiagen, Duesseldorf, Germany). DNA methylation level of *SOX17* gene was defined as “high” if the methylation level of tumor was higher than or equal to the resulting threshold of 31% derived from the average of 70 normal tissues. Primers for pyrosequencing are listed in Additional file 1: Table S1.

### Quantitative reverse transcriptase-polymerase chain reaction (RT-qPCR) and chromatin-immunoprecipitation-polymerase chain reaction (ChIP-qPCR) assays

RT-qPCR was performed to detect the mRNA expression level of *SOX17,* DNA repair-related and damage-responsive genes using the StepOnePlus Real-Time PCR System (Applied Biosystems, Carlsbad, CA, USA) with *β-actin* as internal control. The cDNA of samples were amplified using the Fast SYBR Green Master Mix (Applied Biosystems, Carlsbad, CA, USA). The concentration ratios of target genes were calculated using 2^-⊿Ct^ (C_t-*genes*_ - Ct-_*β-actin*_). Data were presented as fold differences relative to target gene expression in tumor versus matched-normal tissues based on calculations of 2^-⊿⊿Ct^ (⊿⊿Ct = ⊿C_t-tumor_ - ⊿C_t-normal_). The low expression of target gene *SOX17* mRNA was defined as the 2^-⊿⊿Ct^ value lower than 1. Cells (1 × 10^7^ cells) were cross-linked with 1% formaldehyde for 10 min at 37 °C, followed by preparation of nuclear lysates using Magna ChIP™ protein G Kit (Millipore Co., Billerica, MA, USA). Chromatin was immunoprecipitated with SOX17 antibody and normal IgG (negative control) using the condition described in Additional file 1: Table S2. The DNA samples recovered from ChIP were analyzed by quantitative real time PCR. The total input served as internal control. PCR primer sequences and annealing temperature are listed in Additional file 1: Table S1.

### Western blot analysis

Cells were lysed on ice using the RIPA buffer. Lysates were then centrifuged at 13,000 r.p.m. for 15 min, 4 °C. Protein extracts were solubilized in sodium dodecyl sulfate (SDS) gel loading buffer (60 mM Tris base, 2% SDS, 10% glycerol, 5% β-mercaptoethanol). Samples containing equal amounts of protein were separated on an 8% SDS-PAGE and electroblotted onto Immobilon-P membranes (Millipore) in a transfer buffer. Membranes were blocked with 5% skim milk in phosphate-buffered saline tween (PBST) (1 × PBS, 0.1% Tween-20) for 1 h at room temperature. Immunoblotting was performed using antibodies against SOX17, BRCA1, RAD51, KU80, DNAPK, p21 and SIRT1. β-actin was used as loading control. Immunoreactive proteins were visualized using Western blot Enhanced ChemiLuminescent reagents and luminescence protein signals were detected by Luminescence Readers (FUJI LAS-1000, Tokyo, Japan). Each Western analysis was repeated at least three times. The antibodies and blotting conditions are listed in Additional file 1: Table S2.

### Cell viability assay

Cells were seeded at 5 × 10^4^ cells/well in 6-well plates and maintained for 14~16 h. For CCRT sensitization study, cells were treated with radiation (0.5, 1 or 2 Gy) or various concentrations (0.1, 0.25, 0.5, 1 or 2.5 μM) of chemotherapeutic agent cisplatin (Abiplatin® Injection, Pharmachemie, Netherlands) alone or together. Untreated cells served as control. After 72 h treatment, cells were washed with phosphate buffer saline (PBS) and then treated with 0.5 mg/ml of 3-(4.5-dimethylthiazol-2-ly)-2,5-diphenyl tetrazolium bromide (MTT, Sigma-Aldrich). Percentage loss of cell viability compared with control was calculated.

### Foci formation assay

Cells were seeded at 300 cells per well in 6-well plates for 14~16 h and treated with different dose of radiation or various concentrations of cisplatin alone or together for 72 h. Culture media were replaced by drug-free medium and cells were cultured for 6~8 days. Cell colonies were analyzed by ImageJ software. The relative colony formation ability was normalized to control cells (untreated).

### Tumor growth assays in animal model

Three-week old female nude mice were obtained from National Laboratory Animal Center and raised in pathogen-free conditions after obtaining appropriate institutional review board permission. KYSE510-R-EV and KYSE510-R-SOX17 were harvested in Hanks’ balanced salt solution (HBSS). The mice were implanted subcutaneously with 2 × 10^6^ cells in 100 μl HBSS for tumor growth assay. When the tumor volume reached 50 mm^3^, mice were treated with CCRT (2 Gy of radiation on Day 1 and 2 mg/kg cisplatin twice a week for two weeks). Tumor volume was measured on every three days and mice were sacrificed by cervical dislocation on day 35. Tumor samples were resected and weighed prior to 4% formaldehyde fixation. Half of the excised tissues were embedded and sectioned for immunohistochemistry staining. Another half of tissues were subjected to RNA extraction for further mRNA expression analyses.

### Immunohistochemistry (IHC) staining

The protein expression level of SOX17 was evaluated by IHC of endoscopic biopsy from ESCC patients and xenograft tissues from mice. For xenograft tissues, the protein expression of BRCA1, p21 and SIRT1 were also examined by IHC. The evaluation of IHC was conducted blindly without knowledge of the clinical and pathologic characteristics of the patients. The surrounding non-neoplastic stroma served as internal control for each slide.

TissueGnostics FACS-like Tissue Cytometry (TissueGnostics, Vienna, Austria) was used to quantify SOX17 IHC staining of tumor region on endoscopic biopsy slides. The staining “mean intensity of DAB” measurements were translated into continuous value. The staining “percentage” measurements were graded using 0–100 percentage system. The IHC data were defined by “percentage x mean intensity of DAB”. Same definition was applied to IHC of other proteins in tumor xenografts derived from mice. The IHC conditions are listed in Additional file 1: Table S2.

### Statistical analysis

The statistical analyses of promoter methylation, RNA expression, and patients’ CCRT response were performed using Statistical Package for the Social Sciences version 17.0 (SPSS Inc., Headquarters Chicago, IL, USA). The computation of confidence intervals of areas under the curve (AUC) and the multivariate logistic regression analyses were conducted. Pearson χ^2^ test was used to compare the correlation of mRNA and DNA methylation and clinicopathological parameters in ESCC patients. Two-tailed Student’s *t*-test was used in cell and animal studies. Each experiment was repeated at least three times and represented as mean ± S.D. *P* < 0.05 was considered as statistically significant.

## Results

### Low expression of SOX17 mRNA and protein along with promoter hypermethylation correlates with CCRT response in ESCC patients

**Fig. 1:**
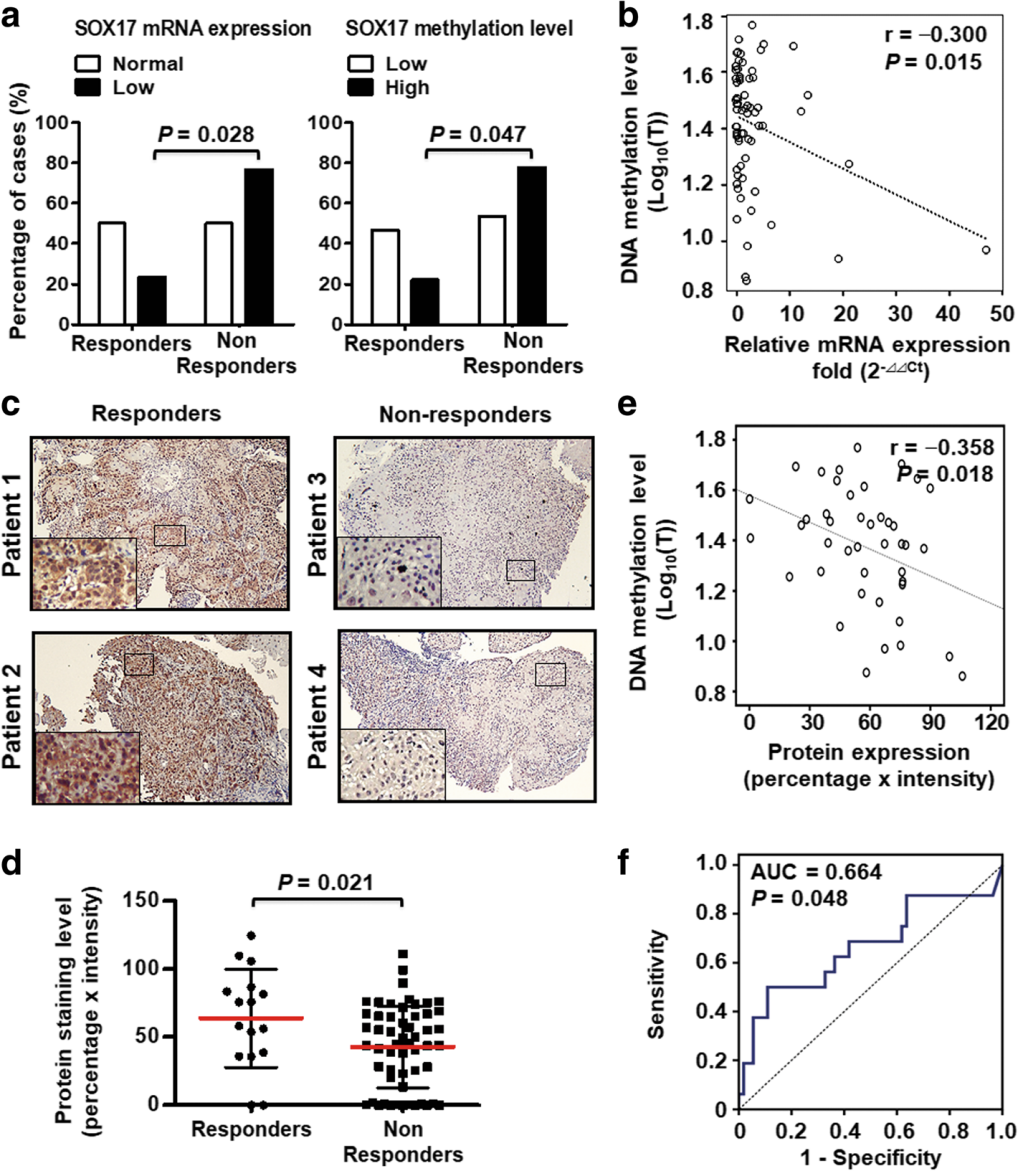
SOX17 mRNA expression, DNA methylation and protein expression correlate with CCRT response in 70 ESCC patients. **a** Concordance analysis of low *SOX17* mRNA expression and high *SOX17* methylation in non-responders ESCC patients. The percentage of ESCC cases is indicated in the plot. *P*-value was calculated by Pearson χ2-test. **b** Inverse correlation between DNA methylation and mRNA expression in *SOX17* gene. DNA methylation level of tumor (Log_10_(T)) tissues is indicated in the Y-axis, while tumor mRNA expression (Log_10_(T)) is plotted as the X-axis. One circle symbolizes one patient sample. Correlation coefficients (r) and *P* value were calculated by Pearson correlation coefficient analysis and are shown at the top. **c** Representative IHC of SOX17 protein in endoscopic biopsy sample from four ESCC patients. SOX17 nuclear immunoreactivity (+) was found in responder patients 1 and 2, whereas non-responder patients 3 and 4 showed SOX17 expression (−) in nuclei of tumor tissue. A 4-fold enlarged image of tumor area indicated by black box is shown in lower left inset for each patient (Original magnification: 100X). **d** IHC staining results showed higher SOX17 expression in CCRT responders than CCRT non-responders. The SOX17 IHC staining level is indicated in the Y-axis. One dots represented one patient slides. *P* values were calculated by 2-tailed *t* test and are shown as indicated. **e** Inverse correlation between DNA methylation and protein expression in SOX17. **f** ROC curve showing the performance of SOX17 protein expression in predicting the CCRT response, with the area under curve (AUC) being 0.664 and *P* values being 0.048

We previously reported that *SOX17* promoter hypermethylation resulted in low expression of its mRNA and protein, and correlated with poor prognosis in ESCC patients [26]. However, the role of SOX17 in sensitization of therapeutic response has never been reported. We therefore investigated whether SOX17 expression and gene methylation correlated with CCRT response in ESCC patients. A total of 70 pre-treatment endoscopic specimens from ESCC patients who received CCRT with follow-up of response status were collected for analysis. We examined the mRNA expression level of *SOX17* by RT-qPCR and the methylation level by pyrosequencing. The results indicated that 76.5% of CCRT non-responders showed low *SOX17* mRNA expression. In contrast, low *SOX17* mRNA expression was only found in 23.5% in CCRT responders (*P =* 0.028, Fig. 1a, *left*). Importantly, 77.8% of CCRT non-responders showed *SOX17* DNA hypermethylation, whereas only 22.2% of CCRT responders showed *SOX17* DNA hypermethylation (*P* = 0.047, Fig. 1a, *right*). The data suggested that low *SOX17* mRNA expression and DNA hypermethylation significantly correlated with poor CCRT response. In addition, DNA methylation and mRNA expression of SOX17 displayed significant inverse correlation (r = − 0.300, *P* = 0.015, Fig. 1b).

Next, we examined the protein expression level of SOX17 in tissue slides of 70 pre-treatment endoscopic samples by IHC staining. The SOX17 protein expression was scored as stained intensity × percentage of tumor regions of endoscopic samples (Fig. 1c). In addition, IHC staining results demonstrated lower SOX17 protein expression in CCRT non-responders compared with CCRT responders (*P* = 0.021, Fig. 1d). The IHC results indicated an inverse correlation between the DNA methylation and protein expression of SOX17 (*r* = − 0.358, *P* = 0.018, Fig. 1e), suggesting that DNA hypermethylation of *SOX17* resulted in low protein expression in ESCC patients. ROC curve analysis was utilized to analyze the sensitivity and specificity of SOX17 protein expression as a prediction biomarker for CCRT responses. The AUC analysis (0.664, *P* = 0.048) (Fig. 1f) indicated that low SOX17 protein expression was associated with poor CCRT response in ESCC patients.

### Stable overexpression of SOX17 sensitizes ESCC cell line to cisplatin, radiation and chemoradiation treatments

**Fig. 2:**
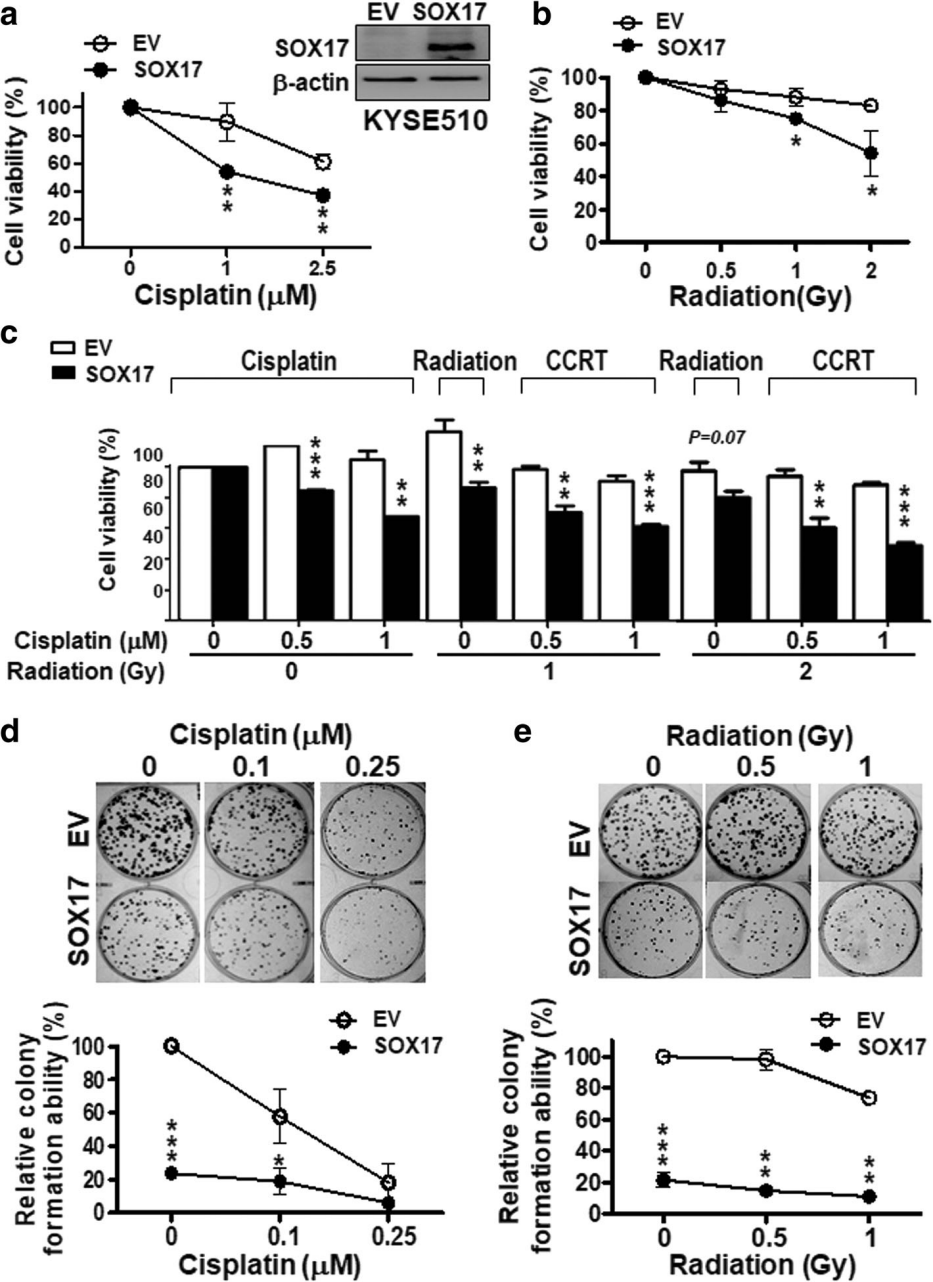
Stable overexpression of *SOX17* sensitizes ESCC cell line to cisplatin, radiation and chemoradiation treatments. **a-c** Cell viability was measured by MTT assay in KYSE510-SOX17 stable cells treated with various doses of cisplatin (**a**), radiation (**b**), or CCRT (concurrent cisplatin and radiation treatment) (**c**) for 72 h. KYSE510-SOX17 stable cells were more sensitive to treatments described above than KYSE510-EV (empty vector) cells. Western blot analysis confirmed that SOX17 protein was stably expressed in KYSE510-SOX17 cell, but not in KYSE510-EV cell (upper right panel in **a**). **d**, **e** Clonogenic survival was measured at day 6 after replating the KYSE510-SOX17 or KYSE510-EV cells treated with different doses of cisplatin (**d**) or radiation (**e**) for 72 h. The foci formation ability of KYSE510-SOX17 stable cells were reduced compared to KYSE510-EV cells. Data are mean ± S.D. from three independent experiments. *P*-values were determined by two-tailed Student’s *t*-test. *P < 0.05; **P < 0.01; ***P < 0.001

Since low expression of SOX17 occurred in CCRT non-responder ESCC patients, we next examined whether expression of SOX17 could increase sensitivity of ESCC cells to anti-cancer therapies. We established a stable KYSE510 cell line expressing SOX17, KYSE510-SOX17 (Fig. 2a, *upper right panel*), and then tested the effects of SOX17 overexpression on clonogenic survival upon cisplatin, radiation and chemoradiation (combination treatment of cisplatin and radiation, CCRT) treatments by MTT assay and foci formation assay. KYSE510-SOX17 stable cells displayed a decrease in cell viability compared with KYSE510-EV (empty vector) control cells when treated with various doses of cisplatin, radiation, and CCRT for 72 h (Fig. 2a-c). Similar results were observed for the clonogenic survival by foci formation assay, showing that KYSE510-SOX17 stable cells with abundant SOX17 expression had fewer colonies when exposed to different doses of cisplatin or radiation compared to KYSE510-EV cells (Fig. 2d, e). However, si-knockdown of SOX17 in KYSE510 cells led to an increase in cell viability upon cisplatin treatment as compared to si-control cells. The enhanced cell viability by knocking down of SOX17 upon cisplatin treatment was confirmed in an addition cell line KYSE170 (Additional file 1: Figure S1). Thus, these results indicated that SOX17 overexpression likely contributed to the increased sensitivity of ESCC cells to cisplatin, radiation and CCRT treatments.

### ESCC radio-resistant cell line shows low *SOX17* mRNA expression with DNA hypermethylation

**Fig. 3:**
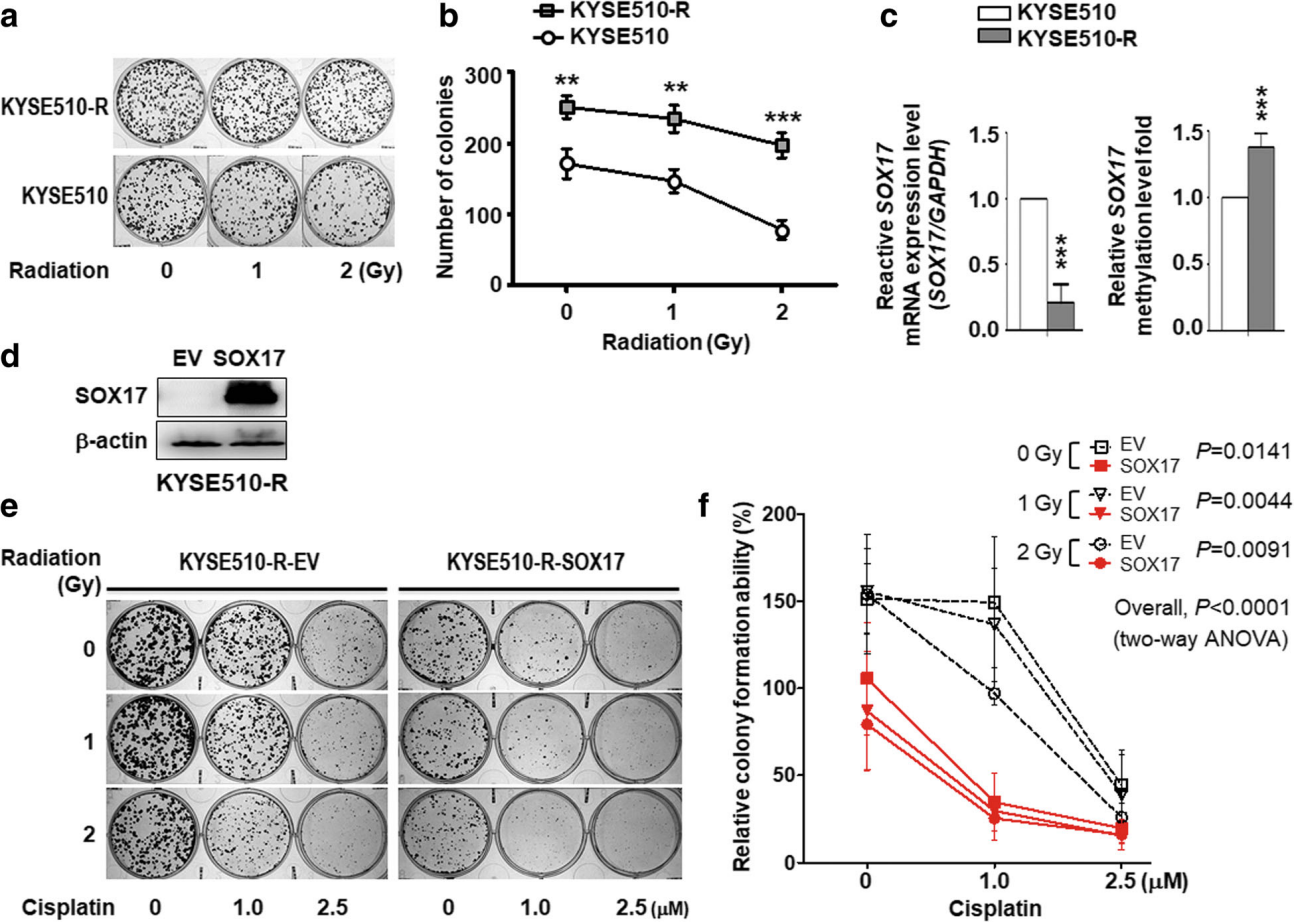
Overexpression of SOX17 sensitized ESCC radiation resistant cell line to CCRT treatment. **a** Cells were treated with radiation (0~2 Gy). The foci were stained and scored on day 6 after radiation. **b** KYSE510 radio-resistant (KYSE510-R) cells showed higher clonogenic survival under radiation condition compared with KYSE510 parental (KYSE510) cells. *P* values were calculated by two-tailed Student’s *t* test from three independent experiments. **c** The RT-qPCR results showed that KYSE510-R cells had lower *SOX17* mRNA expression than KYSE510 parental cells (*left*). The DNA pyrosequencing methylation results indicated that KYSE510-R cells had 1.38-fold higher *SOX17* DNA methylation level compared to KYSE510 cells (*right*). *P* values were calculated by two-tailed *t* test. **d** Western blot analysis confirmed that SOX17 protein was expressed in KYSE510-R-SOX17 cell. **e** Clonogenic survival was measured at day 6 after replating the KYSE510-R-SOX17 and KYSE510-R-EV cells treated with different doses of cisplatin (0~2.5 μM), radiation (0~2 Gy), or CCRT for 72 h. **f** The colony formation ability of KYSE510-R-SOX17 cells were inhibited compared to KYSE510-R-EV cells after CCRT treatment with 1 μM of cisplatin and 1 Gy of radition. *P* values were calculated by two-way ANOVA. *P*-values were determined by two-tailed Student’s *t*-test. *P < 0.05; **P < 0.01; ***P < 0.001

To further validate the role of SOX17 in sensitization of ESCC to chemoradiation, we established KYSE510 radio-resistant cell line (KYSE510-R), and performed the foci formation assay to confirm radio resistance under radiation treatment (Fig. 3a). KYSE510-R cell formed larger and more number of cell colonies compared with KYSE510 parental cell, indicating that KYSE510-R cell indeed had the higher capability to survive under radiation environment (Fig. 3b). Importantly, *SOX17* mRNA expression was significantly lower in KYSE510-R cells than KYSE510 parental cells (Fig. 3c, *left*), while KYSE510-R cells showed 1.38-fold higher *SOX17* methylation level than KYSE510 parental cells (Fig. 3c, *right*). These results suggested that low *SOX17* mRNA expression occurred in radio-resistant ESCC cells in part due to hypermethylation of *SOX17* promoter.

### Overexpression of SOX17 sensitized radio-resistant ESCC cell line to cisplatin, radiation and chemoradiation treatments

Since low expression of *SOX17* occurred in ESCC radio-resistant cells, we next examined whether re-expression of *SOX17* could sensitize their responses to anti-cancer treatments. We established a stable *SOX17* expression KYSE510 radio-resistant cell line, KYSE510-R-SOX17 (Fig. 3d), and then tested the effects of SOX17 overexpression on clonogenic survival to cisplatin, radiation and CCRT treatments. KYSE510-R-SOX17 cells were treated with various doses of cisplatin, radiation, and CCRT for 72 h, and then subjected to foci formation assay to assess clonogenic viability. KYSE510-R-SOX17 cells with abundant SOX17 expression had fewer and smaller colonies when exposed to different doses of radiation, cisplatin, and CCRT compared with KYSE510-R-EV radio-resistant cells (Fig. 3e-f, *P* < 0.0001, two-way ANOVA). Together, these results indicated that SOX17 re-expression sensitized ESCC radio-resistant cells to cisplatin, radiation and CCRT treatments.

### Increased expression of DNA repair and DNA damage response genes in ESCC radio-resistant cell line

**Fig. 4:**
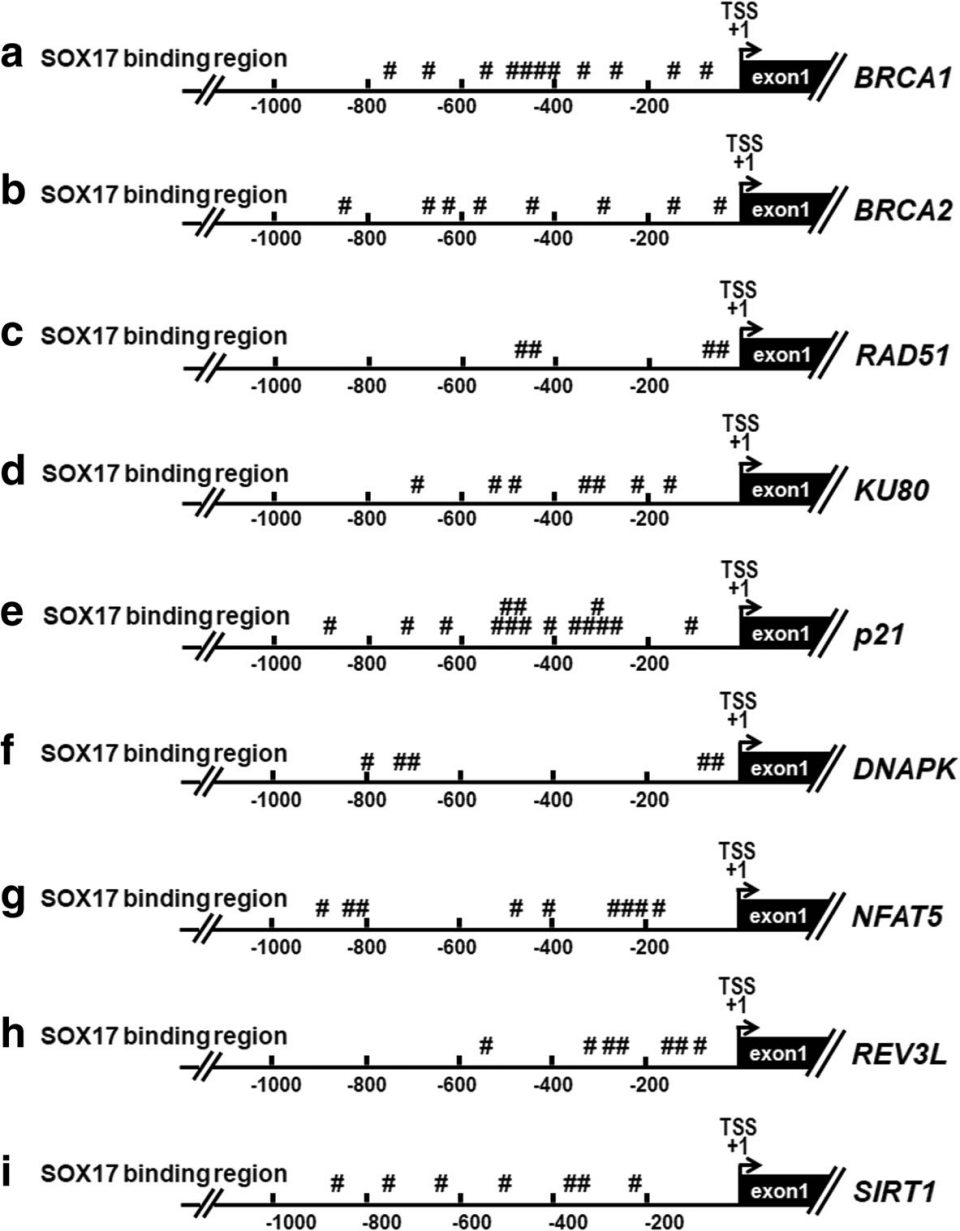
The genomic maps of SOX17 binding sites in promoter of DNA repair genes and DNA damage response genes analyzed in the current study. Hashtags (#) indicate the SRY sites (5′-(A/T)(A/T)CAA(A/T)G-3′) in promoter region (− 1000 ~ + 1), which are the binding site for SOX17 and are predicted using PROMO software. TSS: transcription start site as indicated by (+ 1). The nucleotides relative to TSS are shown

**Fig. 5:**
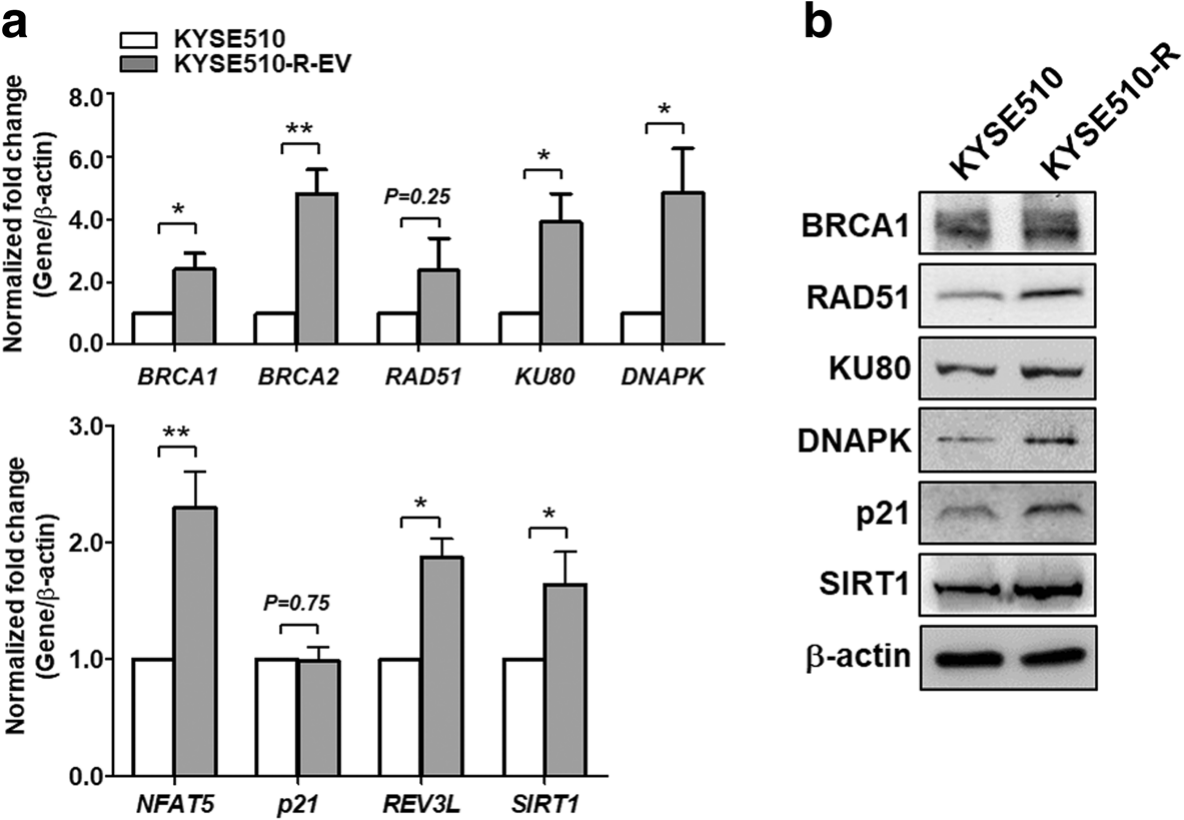
Increased expression of DNA repair and DNA damage response genes in ESCC radio-resistant cell line. **a** RT-qPCR showed that KYSE510 radio-resistant cells (KYSE510R) exhibited significantly higher basal expression levels of DNA repair genes (*upper*) and DNA damage response genes (*lower*) in comparison with KYSE510 parental cells. Data represent mean ± S.D. *P*-values were determined by two-tailed Student’s *t*-test. *P < 0.05; **P < 0.01. **b** The protein expression level of DNA repair proteins and DNA damage response proteins in KYSE510-R was higher than that in KYSE510 cells. β-actin was used as an internal control for western blots

Next, we investigated the mechanism underlying SOX17-mediated sensitization of cisplatin, radiation and CCRT treatments. Studies have reported that enhancement of DNA repair is crucial for the development of resistance, and defects in DNA repair pathways caused increase sensitivity to DNA-damaging agents such as chemotherapeutic agents and radiation in tumor cells [30, 31]. For example, homologous recombination (HR) pathway and non-homologous end joining (NHEJ) pathway were responsive to DNA double strand breaks, which could be induced by radiation, UV light, and chemotherapy agent cisplatin [32]. Therefore, we hypothesized that SOX17 would transcriptionally down-regulate DNA repair-related genes and damage-responsive genes to sensitize ESCC cells to anti-cancer treatments. We found several putative SOX17 binding sites in promoter regions (− 1000 ~ + 1) of genes involved in HR pathway, such as *BRCA1* (*breast cancer 1, early onset*), *BRCA2* (*breast cancer 2, early onset*), *RAD51* (*RAD51 homolog (S. cerevisiae)*), and NHEJ pathway, including *KU80* (*X-ray repair complementing defective repair in Chinese hamster cells 5 (double-strand-break rejoining)*) and *DNAPK* (*protein kinase, DNA-activated, catalytic polypeptide*). In addition, SOX17 binding sites were found in genes involved DNA damage response including *NFAT5* (*Nuclear factor of activated T-cells 5, tonicity-responsive*), *p21* (*cyclin-dependent kinase inhibitor 1A*), *REV3L* (*REV3-like, DNA polymerase zeta catalytic subunit directed*) and *SIRT1* (*sirtuin 1*) (Fig. 4; Additional file 1: Table S3). Therefore, we examined the mRNA and protein expression levels of DNA repair and DNA damage response genes in ESCC radio-resistant KYSE510-R cells and the parental KYSE510 cells. The results showed that mRNA and protein expression levels of these genes were mostly up-regulated in the KYSE510-R compared with parental KYSE510 cells (Fig. 5a, b). These findings suggested that increased expression of DNA repair and DNA damage response genes may account for radio-resistance in KYSE510-R cells.

### Overexpression of *SOX17* decreases mRNA expression and promoter activity of DNA damage responsive and repair genes to sensitize ESCC cells to anti-cancer treatments

**Fig. 6:**
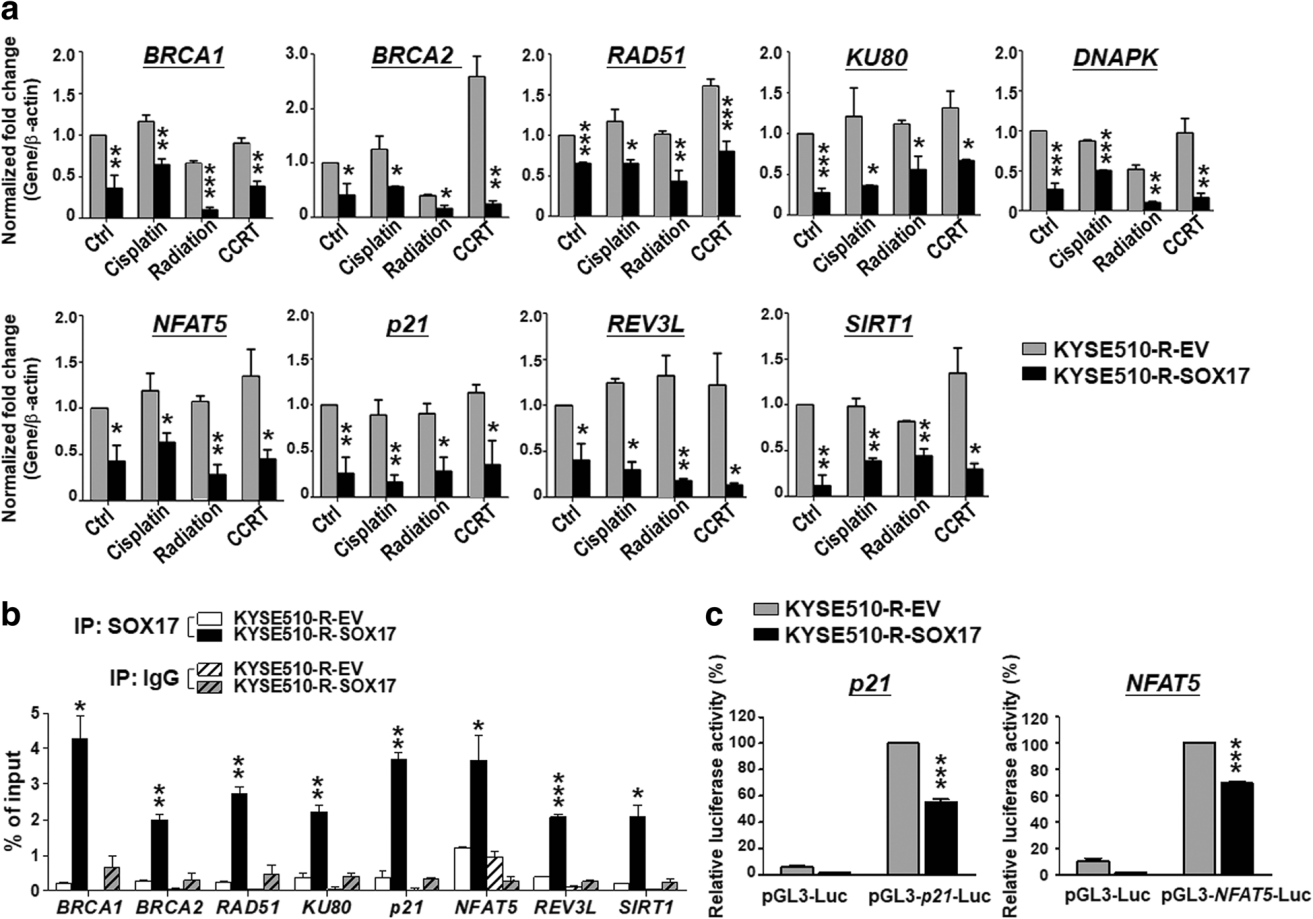
SOX17 overexpression suppresses DNA repair and damage response genes though transcriptional regulation. **a** KYSE510 radio-resistant cells (KYSE510-R-EV and KYSE510-R-SOX17) were treated with cisplatin 1 μM, radiation 2 Gy, or CCRT treatments. After 24 h, cells were harvested and analyzed for mRNA expression level by qRT-PCR. mRNA expression levels of DNA repair genes (*upper*) DNA damage response genes (*lower*) were inhibited in KYSE510-R-SOX17 cells in comparison with KYSE510-R-EV cells. **b** ChIP-qPCR showed that SOX17 bound to the promoter region of genes analyzed in KYSE510-R-SOX17 cells (*black bars*). ‘IgG’ used as a negative control (*gray bars*). **c** SOX17 downstream genes *p21* and *NFAT5* promoter activities were significantly inhibited in KYSE510-R-SOX17 compared to KYSE510-R-EV cells. Data represent mean ± S.D. from three independent experiments. *P*-values were determined by two-tailed Student’s *t*-test. *P < 0.05; **P < 0.01; ***P < 0.001

To investigate whether SOX17 sensitizes ESCC cells to anti-cancer treatments by transcriptionally down-regulating DNA damage-responsive and repair-related genes, we examined the effect of SOX17 overexpression on mRNA expression of these genes in KYSE510-R-SOX17 cells treated with cisplatin, radiation, or CCRT. Notably, RT-qPCR results showed that mRNA levels of DNA repair genes *BRCA1*, *BRCA2*, *RAD51*, *KU80* and *DNAPK* (Fig. 6a, *upper*) as well as DNA damage-responsive genes *p21, SIRT1, NFAT5* and *REV3L* (Fig. 6a, *lower*) were markedly reduced in KYSE510-R-SOX17 cells compared to KYSE510-R-EV cells. In addition, overexpression of SOX17 reduced the protein expression level of DNA repair and damage-responsive genes analyzed (Additional file 1: Figure S2). Together these mRNA and protein results indicated that SOX17 overexpression sensitized ESCC resistant cells to anti-cancer treatment via down-regulation of DNA repair or damage-responsive genes.

To further confirm whether SOX17 regulated transcriptional activity of its downstream genes, we performed ChIP-qPCR in KYSE510-R-SOX17 cells and KYSE510-R-EV cells. ChIP-qPCR showed the significantly enriched binding of SOX17 at *BRCA1, BRCA2, RAD51, KU80, p21, NFAT5*, *REV3L* and *SIRT1* promoters in KYSE510-R-SOX17 cells as compared to that in KYSE510-R-EV cells (Fig. 6b). Furthermore, we conducted promoter activity assay using *p21 promoter*-Luciferase reporter or *NFAT5 promoter*-Luciferase reporter plasmid. Both *p21* and *NFAT5* promoter activities were significantly inhibited in KYSE510-R-SOX17 than KYSE510-R-EV control cells (*P* < 0.001, Fig. 6c). Together, these results suggested that SOX17 may sensitize cells to anti-cancer treatments through in vivo binding to the promoter and thus transcriptional down-regulation of DNA repair genes and damage responsive genes.

### Overexpression of the SOX17 sensitizes ESCC radio-resistant cells to CCRT treatment in xenograft animal models

**Fig. 7:**
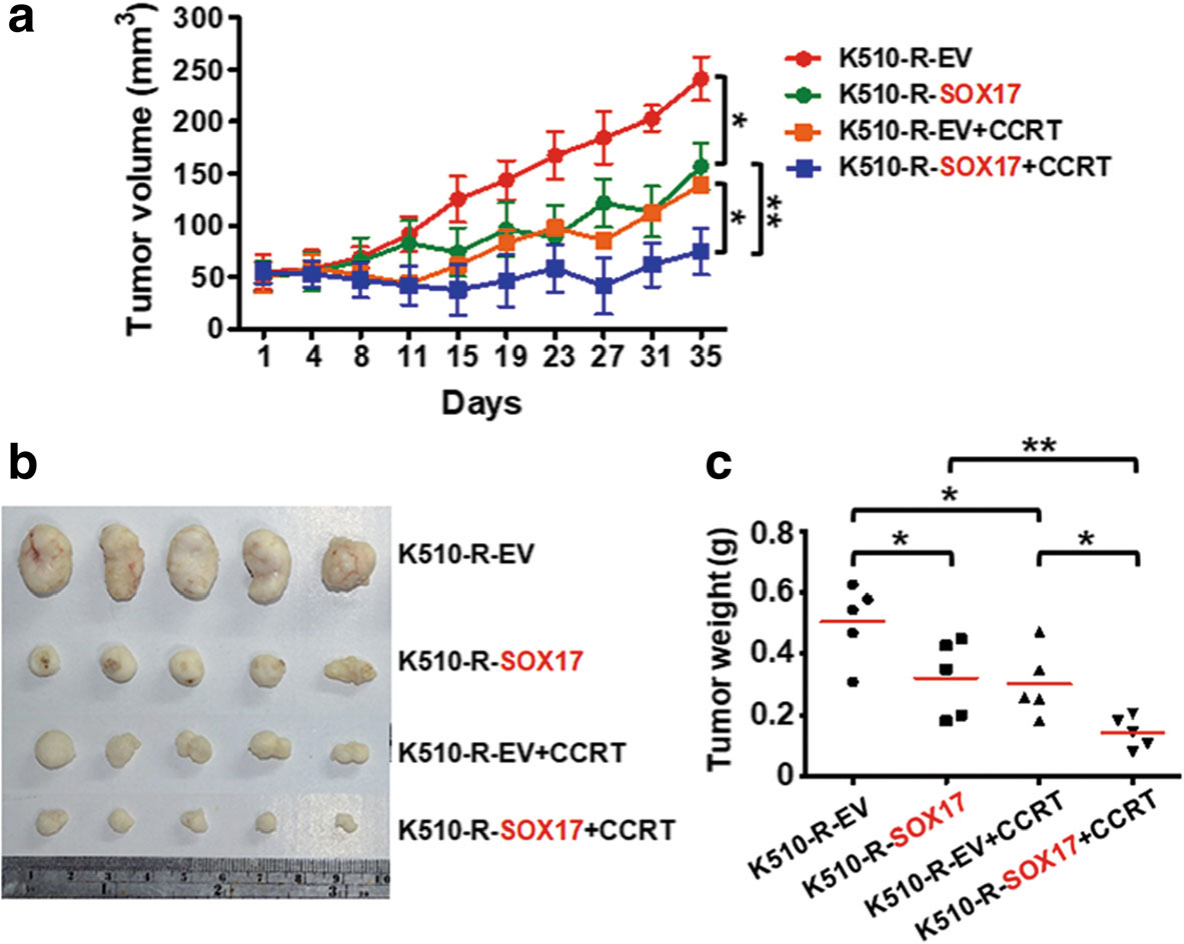
Overexpression of SOX17 reduces tumor growth of xenograft derived from radiation resistant ESCC cell line to CCRT treatment. **a** SOX17 overexpression sensitized tumor xenograft derived from KYSE510 radio-resistant cells (K510-R-SOX17) to CCRT treatment compared with K510-R-EV in ESCC xenograft model. K510-R-EV or K510-R-SOX17 cells were subcutaneously injected into BLAB/c nude mice and treated with CCRT (2 Gy radiation + 2 mg/kg cisplatin as described in text). Mice were observed for tumor volume and sacrificed after 35 days. **b** Xenograft images are shown. **c** SOX17 effectively promoted K510-R cells to reduce tumor weight upon CCRT treatment compared with K510-EV in BLAB/c nude mice (*n* = 5 mice per group)

To confirm the in vivo sensitization effect to CCRT treatment by re-expression of SOX17, we performed the experiments in animal model. KYSE510-R-EV and KYSE510-R-SOX17 cells were subcutaneously injected into nude mice. Mice were treated with 2 Gy of radiation once and 2 mg/kg cisplatin twice per week for two weeks when tumor sizes reached 50 mm^3^. The tumor volume was measured from day 1 to day 35. Tumor samples were resected and weighed on day 35. The results showed that tumor volume (Fig. 7a), tumor size (Fig. 7b) and tumor weight (Fig. 7c) of xenografts in KYSE510-R-SOX17 mice group were decreased in comparison with those in KYSE510-R-EV mice group. Importantly, tumors derived from KYSE510-R-SOX17 were highly sensitive to CCRT treatment in vivo.

### SOX17 overexpression effectively downregulates mRNA expression and protein expression of DNA repair or damage responsive genes in xenograft tissues after CCRT treatment

**Fig. 8:**
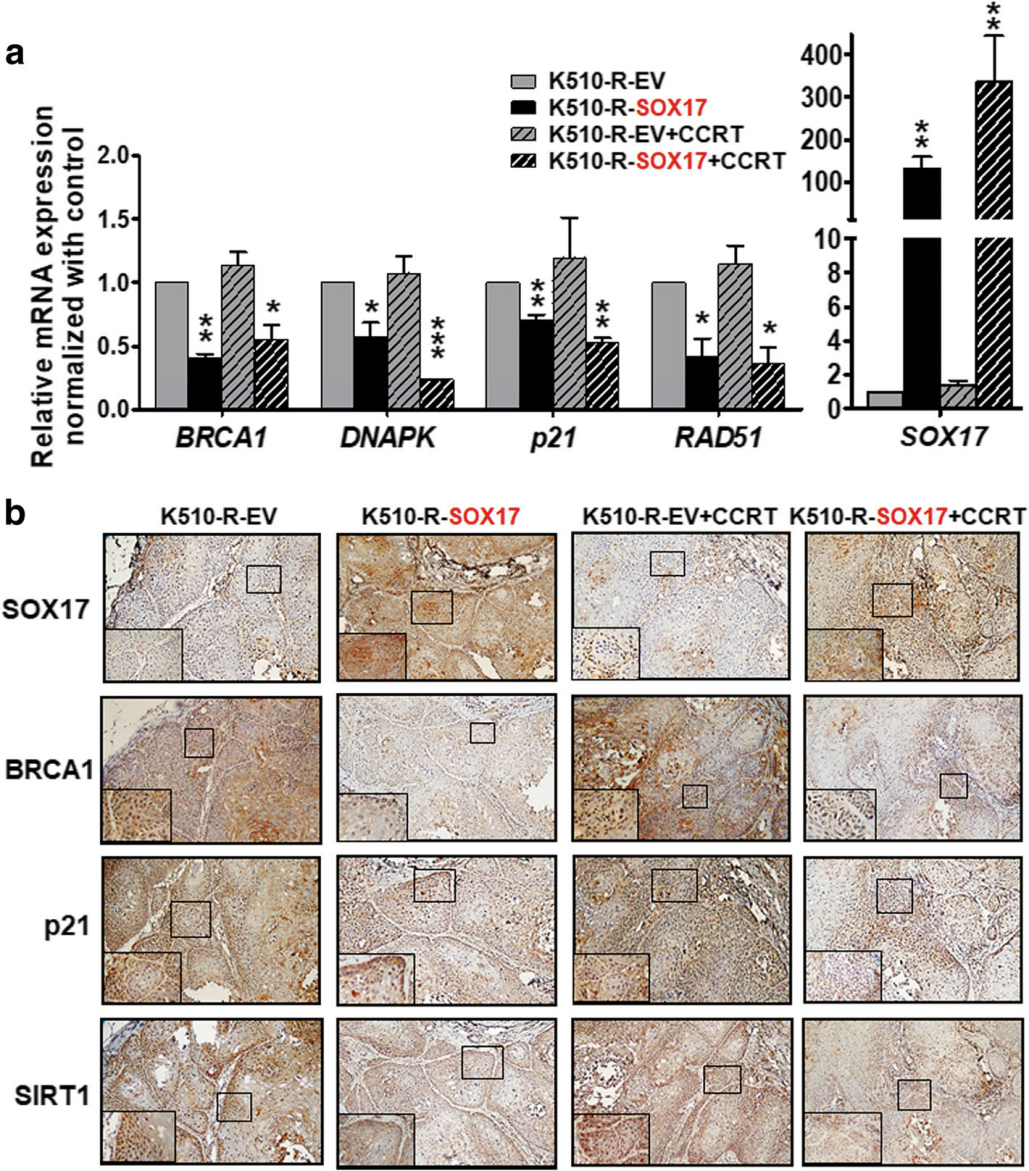
Overexpression of SOX17 suppresses DNA repair genes of xenograft derived from radiation resistant ESCC cell line to CCRT treatment. **a** Xenograft tissues were extracted for RNA and examined for mRNA expression of DNA repair genes and damage response genes. The RT-qPCR results showed that expression of *BRCA1*, *DNAPK*, *p21*, and *RAD51* was inhibited in KYSE510-R-SOX17 cells in comparison with KYSE510-R-EV cells with (*black bar*) or without (*black slashed bar*) CCRT treatment. *SOX17* mRNA levels are shown for comparison (*right*). *P*-values were determined by two-tailed Student’s *t*-test. *P < 0.05; **P < 0.01; ***P < 0.001. **b** Representative IHC results of BRCA1, p21, SIRT1 and SOX17 protein expression in four groups of xenografts. A 4-fold enlarged image of tumor area indicated by black box is shown in lower left inset for each xenograft (Original magnification: 100X)

The results of our experiments using cell model suggested that SOX17 overexpression sensitized ESCC cells to CCRT response by downregulating the mRNA expression of DNA repair and DNA damage response gene. Therefore, we next verified these results in ESCC xenograft model. The RT-qPCR results of xenograft tissues showed that mRNA expression of *BRCA1*, *DNAPK*, *p21* and *RAD51* decreased when SOX17 was overexpressed (KYSE510-R-SOX17 vs. KYSE510-R-EV) and the effects of downregulation were more dramatic in most of genes under CCRT treatment (KYSE510-R-SOX17 + CCRT vs. KYSE510-R-EV + CCRT) (Fig. 8a).

Furthermore, we examined the protein expression of SOX17 and DNA damage responsive genes by IHC staining. IHC results demonstrated that expression level of BRCA1, p21 and SIRT1 proteins was reduced upon SOX17 overexpression and the level remained low upon CCRT (Fig. 8b). These in vivo xenograft data corroborated with the in vitro cell-based results that SOX17 overexpression sensitizes ESCC cells and xenografts to CCRT treatment, in part, by downregulation of DNA repair gene or damage response genes.

## Discussion

**Fig. 9:**
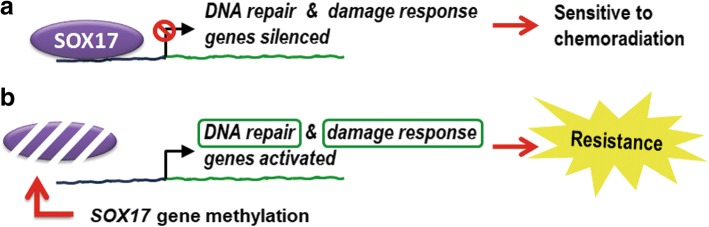
Model of SOX17 molecular functions in sensitization of chemoradiation. **a** Tumor suppressor SOX17 transcriptionally inactivates DNA repair and DNA damage responsive genes to enhance sensitivity to chemoradiation in ESCC. **b** Low SOX17 protein expression resulting from gene hypermethylation is associated with resistance to chemoradiation in ESCC. Low SOX17 immunoreactivity could be a biomarker for CCRT response prediction in pre-treatment endoscopic biopsies from ESCC patients

Despite the recent improvements in diagnostic techniques and multimodality treatments, most ESCC patients develop resistance to therapy resulting in treatment failure. In the present study, we provided a proof-of-concept CCRT prediction biomarker using SOX17 immunohistochemical staining in pre-treatment endoscopic biopsies to identify ESCC patients not responding to CCRT. Re-expression of SOX17 was confirmed to sensitize radio-resistant ESCC cells to radiation, cisplatin or CCRT treatment in cell and animal models. We further demonstrated a mechanism of SOX17 sensitization via negative regulation of DNA repair and damage responsive genes. Our study provides new evidence that tumor suppressor SOX17 transcriptionally inactivates DNA repair and DNA damage responsive genes to enhance sensitivity to CCRT treatment in ESCC (Fig. 9).

Defects in DNA repair pathways have been reported to increase sensitivity to DNA-damaging agents [31, 32]. Here, we reveal a novel mechanism that SOX17 significantly decreased expression of genes involved in HR and NHEJ pathways via transcription regulation. Genes involved in DNA damage response were also downregulated by SOX17. Among them, NFAT5 was reported to facilitate cell proliferation and cell cycle progression in response to hypertonic stress [33]. In addition, reports had implicated that NFAT5 was associated with several DNA repair-related proteins. For example, PARP-1 (poly (ADP-ribose) polymerase) modulated transcriptional activity of NFAT5 [34]. DNA damage response protein ATM (ataxia telangiectasia mutated) was involved in NFAT5 activation in response to hypertonic stress [35]. DNAPK, which plays a central role in NHEJ repair pathway, has been reported to form multiple protein-protein interactions with NFAT5 [34]. Collectively, whether down-regulation of NFAT5 and DNAPK by SOX17 would decrease their complex formation and thus reduced repair activity to increase therapy sensitivity is worthy for further investigations.

Our study provides a proof-of-concept CCRT prediction biomarker, i.e., low SOX17 immunohistochemical stain in pre-treatment endoscopic biopsies that can predict CCRT response in ESCC patients with intrinsic resistance. If possible, SOX17 immunohistochemical stain in endoscopic biopsies obtained during CCRT may identify individuals with acquired resistance. The endoscopic biopsy screening test is a feasible method for close monitoring of clinical outcomes. SOX17 protein expression level could be useful in identifying ESCC patients who is at high risk of CCRT failure and in dire need of early surgery or intensive care. Moreover, SOX17 low expression has been shown to associate with cisplatin or radiation resistance in colorectal cancer, endometrial cancer, or rat mammary carcinoma [36–38], suggesting the pivotal role(s) of SOX17 down-regulation in therapy-resistant cancers.

Notably, low expression of SOX17 protein correlated with high DNA methylation and low mRNA expression in CCRT non-responders, and was confirmed in radio-resistant ESCC cells. Numerous DNA demethylation reagents have been developed and tested in clinical trials as single therapy or in combination with other anti-cancer drugs [39, 40]. The use of DNA demethylation reagents may exert tumor inhibition or sensitization effects through up-regulation of SOX17, inducing potent cytotoxicity in ESCC cells and their resistant cells in multimodality treatments. Further characterization of the SOX17 targeting genes validated in our study could help to dissect the mechanism of ESCC progression and drug resistance.

## Conclusions

In conclusion, our study reveals SOX17 immunoreactivity as a biomarker for CCRT response prediction in pre-treatment endoscopic biopsies from ESCC patients. We provide novel evidence that CCRT sensitization effect of SOX17 in ESCC radio-resistant cell and xenograft models via SOX17 transcriptional regulation of DNA repair and damage response genes. Collectively, our study provides a novel insight into reactivation of tumor suppressive function via SOX17-mediated transcription as critical targets in overcoming resistance in ESCC.

## Additional file

Additional file 1: **Table S1.** The primers used in the current study. **Table S2.** Antibodies and their reaction conditions. **Table S3.** SOX17 binding sites in promoter of DNA repair genes and putative DNA damage response genes in the current study. **Figure S1.** Knockdown of SOX17 sustained the cell viability upon cisplatin treatment in the KYSE510 and KYSE170 cells*.* (A, D) RT-qPCR showed low *SOX17* mRNA expression upon si-knockdown of SOX17 (si-SOX17) in KYSE510 cells (A) or KYSE170 cells (D). (B, E) Western blots confirmed low SOX17 protein expression in si-SOX17 KYSE510 cells (B) or KYSE170 cells (E). β-actin was used as an internal control. (C, F) Knockdown of SOX17 sustained cell viability upon cisplatin treatment measured by MTT assay in si-SOX17 KYSE510 cells (C) or KYSE170 cells (F). The relative cell viability was normalized to si-control (siCtrl) group. Data represent mean ± SD from three independent experiments. *P*-values were determined by two-tailed Student’s *t*-test. *P < 0.05; **P < 0.01; ***P < 0.001. **Figure S2.** Overexpression of SOX17 in KYSE510-R-SOX17 cells reduced the protein expression level of DNA repair and damage-responsive genes analyzed as compared to KYSE510-R-EV cells. β-actin was used as an internal control for western blots. (PDF 688 kb)

## Abbreviations

CCRT: Concurrent chemoradiotherapy
ESCC: Esophageal squamous cell carcinoma
HR: Homologous recombination
NHEJ: Non-homologous end-joining
SOX17: SRY (sex determining region Y)-box 17

## References

[1] Siegel RL, Miller KD, Jemal A (2018). Cancer statistics, 2018. CA Cancer J Clin.

[2] Khuroo MS, Zargar SA, Mahajan R, Banday MA (1992). High incidence of oesophageal and gastric cancer in Kashmir in a population with special personal and dietary habits. Gut.

[3] Enzinger PC, Mayer RJ (2003). Esophageal cancer. N Engl J Med.

[4] Sakaeda T, Yamamori M, Kuwahara A, Nishiguchi K (2009). Pharmacokinetics and pharmacogenomics in esophageal cancer chemoradiotherapy. Adv Drug Deliv Rev.

[5] Chang WL, Lin FC, Yen CJ, Cheng H, Lai WW, Yang HB (2011). Tumor length assessed by miniprobe endosonography can predict the survival of the advanced esophageal squamous cell carcinoma with stricture receiving concurrent chemoradiation. Dis Esophagus.

[6] van Hagen P, Hulshof MC, van Lanschot JJ, Steyerberg EW, van Berge Henegouwen MI, Wijnhoven BP (2012). Preoperative chemoradiotherapy for esophageal or junctional cancer. N Engl J Med.

[7] Stahl M, Stuschke M, Lehmann N, Meyer HJ, Walz MK, Seeber S (2005). Chemoradiation with and without surgery in patients with locally advanced squamous cell carcinoma of the esophagus. J Clin Oncol.

[8] Bedenne L, Michel P, Bouche O, Milan C, Mariette C, Conroy T (2007). Chemoradiation followed by surgery compared with chemoradiation alone in squamous cancer of the esophagus: FFCD 9102. J Clin Oncol.

[9] Di Fiore F, Lecleire S, Rigal O, Galais MP, Ben Soussan E, David I (2006). Predictive factors of survival in patients treated with definitive chemoradiotherapy for squamous cell esophageal carcinoma. World J Gastroenterol.

[10] Chen GZ, Zhu HC, Dai WS, Zeng XN, Luo JH, Sun XC (2017). The mechanisms of radioresistance in esophageal squamous cell carcinoma and current strategies in radiosensitivity. J Thorac Dis.

[11] Kim YT, Park JY, Jeon YK, Park SJ, Song JY, Kang CH (2009). Aberrant promoter CpG island hypermethylation of the adenomatosis polyposis coli gene can serve as a good prognostic factor by affecting lymph node metastasis in squamous cell carcinoma of the esophagus. Dis Esophagus.

[12] Kwon J, Park M, Kim JH, Lee HW, Kang MC, Park JH (2015). Epigenetic regulation of the novel tumor suppressor cysteine dioxygenase 1 in esophageal squamous cell carcinoma. Tumour Biol.

[13] Sung CO, Han SY, Kim SH (2011). Low expression of claudin-4 is associated with poor prognosis in esophageal squamous cell carcinoma. Ann Surg Oncol.

[14] Lee EJ, Lee BB, Han J, Cho EY, Shim YM, Park J (2008). CpG island hypermethylation of E-cadherin (CDH1) and integrin alpha4 is associated with recurrence of early stage esophageal squamous cell carcinoma. Int J Cancer.

[15] Guo XQ, Wang SJ, Zhang LW, Wang XL, Zhang JH, Guo W (2007). DNA methylation and loss of protein expression in esophageal squamous cell carcinogenesis of high-risk area. J Exp Clin Cancer Res.

[16] Kuroki T, Trapasso F, Yendamuri S, Matsuyama A, Alder H, Mori M (2003). Allele loss and promoter hypermethylation of VHL, RAR-beta, RASSF1A, and FHIT tumor suppressor genes on chromosome 3p in esophageal squamous cell carcinoma. Cancer Res.

[17] Fujiwara S, Noguchi T, Takeno S, Kimura Y, Fumoto S, Kawahara K (2008). Hypermethylation of p16 gene promoter correlates with loss of p16 expression that results in poorer prognosis in esophageal squamous cell carcinomas. Dis Esophagus.

[18] Li B, Wang B, Niu LJ, Jiang L, Qiu CC (2011). Hypermethylation of multiple tumor-related genes associated with DNMT3b up-regulation served as a biomarker for early diagnosis of esophageal squamous cell carcinoma. Epigenetics.

[19] Roth MJ, Abnet CC, Hu N, Wang QH, Wei WQ, Green L (2006). p16, MGMT, RARbeta2, CLDN3, CRBP and MT1G gene methylation in esophageal squamous cell carcinoma and its precursor lesions. Oncol Rep.

[20] Guo W, Wang C, Guo Y, Shen S, Guo X, Kuang G (2015). RASSF5A, a candidate tumor suppressor, is epigenetically inactivated in esophageal squamous cell carcinoma. Clin Exp Metastasis.

[21] Zheng Y, Zhang Y, Huang X, Chen L (2011). Analysis of the RUNX3 gene methylation in serum DNA from esophagus squamous cell carcinoma, gastric and colorectal adenocarcinoma patients. Hepato-Gastroenterology.

[22] Alheim K, Corness J, Samuelsson MK, Bladh LG, Murata T, Nilsson T (2003). Identification of a functional glucocorticoid response element in the promoter of the cyclin-dependent kinase inhibitor p57Kip2. J Mol Endocrinol.

[23] Tseng RC, Chang JM, Chen JH, Huang WR, Tang YA, Kuo IY (2015). Deregulation of SLIT2-mediated Cdc42 activity is associated with esophageal cancer metastasis and poor prognosis. J Thorac Oncol.

[24] Jin Z, Olaru A, Yang J, Sato F, Cheng Y, Kan T (2007). Hypermethylation of tachykinin-1 is a potential biomarker in human esophageal cancer. Clin Cancer Res.

[25] Dong W, Shen R, Cheng S (2014). Reduction of TIP30 in esophageal squamous cell carcinoma cells involves promoter methylation and microRNA-10b. Biochem Biophys Res Commun.

[26] Kuo IY, Wu CC, Chang JM, Huang YL, Lin CH, Yan JJ (2014). Low SOX17 expression is a prognostic factor and drives transcriptional dysregulation and esophageal cancer progression. Int J Cancer.

[27] Zhang W, Glockner SC, Guo M, Machida EO, Wang DH, Easwaran H (2008). Epigenetic inactivation of the canonical Wnt antagonist SRY-box containing gene 17 in colorectal cancer. Cancer Res.

[28] Chang WL, Lai WW, Kuo IY, Lin CY, Lu PJ, Sheu BS (2017). A six-CpG panel with DNA methylation biomarkers predicting treatment response of chemoradiation in esophageal squamous cell carcinoma. J Gastroenterol.

[29] Chang WL, Wang WL, Chung TJ, Lin FC, Yen CJ, Lai WW (2015). Response evaluation with endoscopic ultrasound and computed tomography in esophageal squamous cell carcinoma treated by definitive chemoradiotherapy. J Gastroenterol Hepatol.

[30] Zhou BB, Bartek J (2004). Targeting the checkpoint kinases: chemosensitization versus chemoprotection. Nat Rev Cancer.

[31] Zhu Y, Hu J, Hu Y, Liu W (2009). Targeting DNA repair pathways: a novel approach to reduce cancer therapeutic resistance. Cancer Treat Rev.

[32] Postel-Vinay S, Vanhecke E, Olaussen KA, Lord CJ, Ashworth A, Soria JC (2012). The potential of exploiting DNA-repair defects for optimizing lung cancer treatment. Nat Rev Clin Oncol.

[33] Drews-Elger K, Ortells MC, Rao A, Lopez-Rodriguez C, Aramburu J (2009). The transcription factor NFAT5 is required for cyclin expression and cell cycle progression in cells exposed to hypertonic stress. PLoS One.

[34] Chen Y, Schnetz MP, Irarrazabal CE, Shen RF, Williams CK, Burg MB (2007). Proteomic identification of proteins associated with the osmoregulatory transcription factor TonEBP/OREBP: functional effects of Hsp90 and PARP-1. Am J Physiol Renal Physiol.

[35] Irarrazabal CE, Liu JC, Burg MB, Ferraris JD (2004). ATM, a DNA damage-inducible kinase, contributes to activation by high NaCl of the transcription factor TonEBP/OREBP. Proc Natl Acad Sci U S A.

[36] Zhang Y, Jiang F, Bao W, Zhang H, He X (2016). Wang Het al. SOX17 increases the cisplatin sensitivity of an endometrial cancer cell line. Cancer Cell Int.

[37] Kim SB, Bozeman RG, Kaisani A, Kim W, Zhang L, Richardson JA (2016). Radiation promotes colorectal cancer initiation and progression by inducing senescence-associated inflammatory responses. Oncogene.

[38] Daino K, Nishimura M, Imaoka T, Takabatake M, Morioka T, Nishimura Y (2018). Epigenetic dysregulation of key developmental genes in radiation-induced rat mammary carcinomas. Int J Cancer.

[39] Fahy J, Jeltsch A, Arimondo PB (2012). DNA methyltransferase inhibitors in cancer: a chemical and therapeutic patent overview and selected clinical studies. Expert Opin Ther Pat.

[40] Appleton K, Mackay HJ, Judson I, Plumb JA, McCormick C, Strathdee G (2007). Phase I and pharmacodynamic trial of the DNA methyltransferase inhibitor decitabine and carboplatin in solid tumors. J Clin Oncol.

