# Supplementary material for: SOX17 overexpression sensitizes chemoradiation response in esophageal cancer by transcriptional down-regulation of DNA repair and damage response genes
Source: J Biomed Sci. 2019 Feb 18;26:20. doi: 10.1186/s12929-019-0510-4 (PMC6378712; doi:10.1186/s12929-019-0510-4)
Supplement: Supplementary file 1 — Table S1. The primers used in the current study. Table S2. Antibodies and their reaction conditions. Table S3. SOX17 binding sites in promoter of DNA repair genes and putative DNA damage response genes in the current study. Figure S1. Knockdown of SOX17 sustained the cell viability upon cisplatin treatment in the KYSE510 and KYSE170 cells. (A, D) RT-qPCR showed low SOX17 mRNA expression upon si-knockdown of SOX17 (si-SOX17) in KYSE510 cells (A) or KYSE170 cells (D). (B, E) Western blots confirmed low SOX17 protein expression in si-SOX17 KYSE510 cells (B) or KYSE170 cells (E). β-actin was used as an internal control. (C, F) Knockdown of SOX17 sustained cell viability upon cisplatin treatment measured by MTT assay in si-SOX17 KYSE510 cells (C) or KYSE170 cells (F). The relative cell viability was normalized to si-control (siCtrl) group. Data represent mean ± SD from three independent experiments. P-values were determined by two-tailed Student’s t-test. *P < 0.05; **P < 0.01; ***P < 0.001. Figure S2. Overexpression of SOX17 in KYSE510-R-SOX17 cells reduced the protein expression level of DNA repair and damage-responsive genes analyzed as compared to KYSE510-R-EV cells. β-actin was used as an internal control for western blots. (PDF 688 kb) [file 12929_2019_510_MOESM1_ESM.pdf]

Additional file 1

SOX17 overexpression sensitizes chemoradiation response in esophageal cancer by transcriptional down-regulation of DNA repair and damage response genes

I-Ying Kuo[1,2], Yu-Lin Huang[1], Chien-Yu Lin[1], Chien-Hsun Lin[1],Wei-Lun Chang[3], Wu-Wei Lai[4]* and Yi-Ching Wang[1,2]*

[1]Department of Pharmacology, College of Medicine, National Cheng Kung University, Tainan 701, Taiwan

[2]Institute of Basic Medical Sciences, College of Medicine, National Cheng Kung University, Tainan 701, Taiwan

[3]Department of Internal Medicine, National Cheng Kung University Hospital, College of Medicine, National Cheng Kung University, Tainan 701, Taiwan

[4]Department of Surgery, National Cheng Kung University Hospital, College of Medicine, National Cheng Kung University, Tainan 701, Taiwan

Table S1. The primers used in the current study.

Gene	Primer	5'→ 3' Sequences	Application [a]	PCR size (bp)	Tm (℃)
SOX17 gene_ (+131~+169)	Biotin label-Forward	5'-GGG ACA CCG CTG ATC GTT TAG GGT TTT GGG TAA GTT GTA GAT TAG AT-3'	Pyrosequencing - PCR	172	56-
	Reverse	5'-ACC TAC CCC CCT CCC CTC AA-3'			
	Sequencing	5'-TCC CCT CAA CCC TCC-3'	Pyrosequencing	-- [b]	-- [b]
SOX17 mRNA	Forward	5'-ACG CTT TCA TGG TGT GGG CTA AG-3'	RT-qPCR	113	60
	Reverse	5'-GTC AGC GCC TTC CAC GAC TTG-3'			
BRCA1 mRNA	Forward	5'-TTG CAG TGT GGG AGA TCA AG-3'	RT-qPCR	168	60
	Reverse	5'-CGC TTC TCA GTG GTG TTC AA-3'			
BRCA2 mRNA	Forward	5'-ACC CAG CTT ACC TTG AGG GTT ATT T-3'	RT-qPCR	185	60
	Reverse	5'-AAT ACG CAA CTT CCA CAC GGT TG -3'			
RAD51 mRNA	Forward	5'-ATG GTC TCT CTG GCA GTG ATG T -3'	RT-qPCR	124	60
	Reverse	5'-AGC AGT GCA TAC CTA GAT TCT ACC A -3'			
KU80 mRNA	Forward	5'-CCC CAA TTC AGC AGC ATA TT-3'	RT-qPCR	157	60
	Reverse	5'-CCT TCA GCC AGA CTG GAG AC-3'			
DNAPK mRNA	Forward	5'-CAT GGA AGA AGA TCC CCA GA -3'	RT-qPCR	155	60
	Reverse	5'-TGG GCA CAC CAC TTT AAC AA -3'			

NFAT5 mRNA	Forward	5’-TGA GGG AAA GGA GCT GAA GA -3’	RT-qPCR	165	55
	Reverse	5’-CCC ACA AAC ACT TGC AAC AC-3”			
p21 mRNA	Forward	5’-TCA CCG AGA CAC CAC TGG AG-3’	RT-qPCR	300	60
	Reverse	5’-TGG AGT GGT AGA AAT CTG TC-3’			
REV3L mRNA	Forward	5’-TTC CGA AAA GCA AGA AGG AA-3’	RT-qPCR	149	60
	Reverse	5’-TGT GGT TCA ACA CCT TCC AA-3’			
SIRT1 mRNA	Forward	5’-GCA GAT TAG TAG GCG GCT TG-3’	RT-qPCR	152	60
	Reverse	5’-TCT GGC ATG TCC CAC TAT CA-3’			
*β-acti*n mRNA	Forward	5’-GGC GGC ACC ACC ATG TAC CCT-3’	RT-qPCR	180	60
	Reverse	5’-AGG GGC CGG ACT CGT CAT ACT-3’			
BRCA1	Forward	5’-CCG CTC TGG TAT TGG ATG TT-3’	ChIP-qPCR	234	60
	Reverse	5’-CGA AGG TCA GAA TCG CTA CC-3’			
BRCA2	Forward	5’-TTG TAA GAT CGG CTC GCT TT-3’	ChIP-qPCR	127	60
	Reverse	5’-TTT GCT CCA GCT CAT GTT TG-3’			
RAD51	Forward	5’-CCG TAC GCT AGC TCC ATT TC-3’	ChIP-qPCR	175	60
	Reverse	5’-AGC GCT CTT GTG GTT TGT TT-3’			
KU80	Forward	5’-AAG CTG GAG CCA CTG CTA AC-3’	ChIP-qPCR	155	60
	Reverse	5’-TTA GAG CCA ACT GGG GAA GA-3’			
NFAT5	Forward	5’-TTC AGC CTC CAA AGG TAG GA-3’	ChIP-qPCR	153	60
	Reverse	5’-CTT TGG GGT TTG CAT AGC AG-3’			

p21	Forward	5'-TGC CAC TGC TGA CTT TGT CT-3'	ChIP-qPCR	176	60
	Reverse	5'-ATA GGG GCA GTC AGC TTT CA-3'			
REV3L	Forward	5'-GAA CTT GAA CCC ACG CTT TC-3'	ChIP-qPCR	177	60
	Reverse	5'-TCC TCT CTG ACT TGG CCA CT-3'			
SIRT1	Forward	5'-GGT GTG AGG AGA GTG GGA AA-3'	ChIP-qPCR	167	60
	Reverse	5'-GTA CAC CTG GCC TGC CTT AG-3'			

[a] RT-qPCR: Quantitative reverse transcription-polymerase chain reaction; ChIP-qPCR: chromatin-immunoprecipitation-polymerase chain reaction.
[b] --: Not applicable.

Table S2. Antibodies and their reaction conditions.

Target	KD	Raised In	Application [a]	Dilution	Source	Catalog No.
β-actin	42	Mouse	Western blot	1:5000	GeneTex	GTX26267
BRCA1	220	Mouse	IHC	1:1000	Santa Cruz	Sc-642
			Western blot	1:1000		
RAD51	37	Rabbit	Western blot	1:3000	Abcam	Ab133534
KU80	80	Mouse	Western blot	1:1000	Neomarkers	MS-332-P-1
DNAPK	450	Rabbit	Western blot	1:1000	Calbiochem	PC127
GAPDH	37	Mouse	Western blot	1:2000	Santa Cruz	Sc-32233
p21	21	Mouse	IHC	1:1200	Santa Cruz	Sc-817
		Rabbit	Western blot	1:1000	Santa Cruz	Sc-397
SIRT1	120	Mouse	IHC	1:1200	Upstate	05-707
			Western blot	1:1000		
SOX17 (clone 2F9)	55	Mouse	IHC	1:100	Origene	TA500045
			Western blot	1:1000		
SOX17	55	Mouse	ChIP	1:1000	R&D	AF1924

[a] IHC: immunohistchemistry; ChIP: chromatin-immunoprecipitation.

Table S3. SOX17 binding sites in promoter of DNA repair genes and putative DNA damage response genes in the current study.

Symbol	Gene Name	Function	Number of SOX17 binding sites[a]
BRCA1	*breast cancer 1, early onset*	Homologous recombination (HR) repair pathway	11
BRCA2	*breast cancer 2, early onset*	Homologous recombination (HR) repair pathway	8
RAD51	*RAD51 homolog (S. cerevisiae)*	Homologous recombination (HR) repair pathway	4
KU80	*X-ray repair complementing defective repair in Chinese hamster cells 5 (double-strand-break rejoining)*	Non-homologous end joining (NHEJ) repair pathway	7
DNAPK	*protein kinase, DNA-activated, catalytic polypeptide*	Non-homologous end joining (NHEJ) repair pathway	5
p21[b]	*cyclin-dependent kinase inhibitor 1A*	DNA damage response, signal transduction by p53 class mediator resulting in cell cycle arrest	15
SIRT1[b]	*sirtuin 1*	Deacetylate histones and non-histone proteins to trigger cell survival in response to stresses and DNA damage	7
NFAT5[b]	*Nuclear factor of activated T-cells 5, tonicity-responsive*	Transcription regulation, response to hypertonic stress and facilitate cell proliferation and cell cycle progression	9
REV3L[b]	*REV3-like, polymerase (DNA directed), zeta, catalytic subunit*	Error-prone translesion DNA synthesis, and trigger cell defense and survival response to stress and DNA damage	7

[a] Number of SOX17-SRY elements (5'-(A/T)(A/T)CAA(A/T)G-3') in promoter region (-1000 ~ +1) were predicted by PROMO software. The genomic map of putative SOX17-SRY binding sites is shown in Figure 4.

Supplementary figure and figure legend

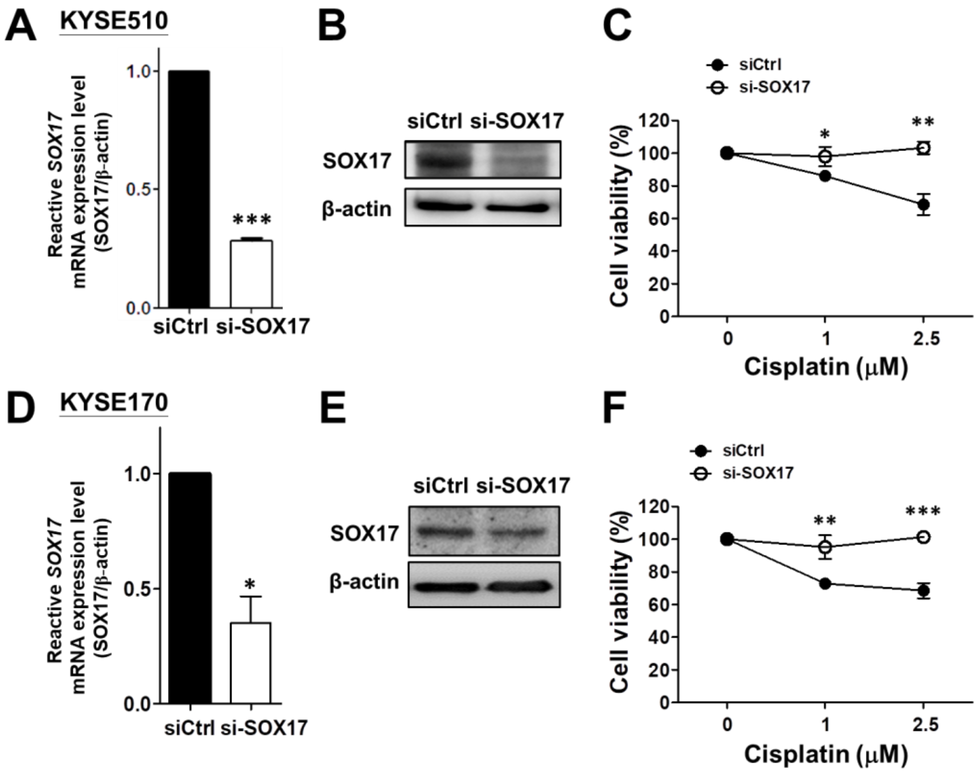

Figure S1 Knockdown of SOX17 sustained the cell viability upon cisplatin treatment in the KYSE510 and KYSE170 cells. (**A, D**) RT-qPCR showed low *SOX17* mRNA expression upon si-knockdown of SOX17 (si-SOX17) in KYSE510 cells (A) or KYSE170 cells (B). (**B, E**) Western blots confirmed low SOX17 protein expression in si-SOX17 KYSE510 cells (B) or KYSE170 cells (E). β-actin was used as an internal control. (**C, F**) Knockdown of SOX17 sustained cell viability measured by MTT assay in si-SOX17 KYSE510 cells (C) or KYSE170 cells (F). The relative cell viability was normalized to si-control (siCtrl) group. Data represent mean ± SD from three independent experiments. *P*-values were determined by two-tailed Student's *t*-test. **P* < 0.05; ***P* < 0.01; ****P* < 0.001.

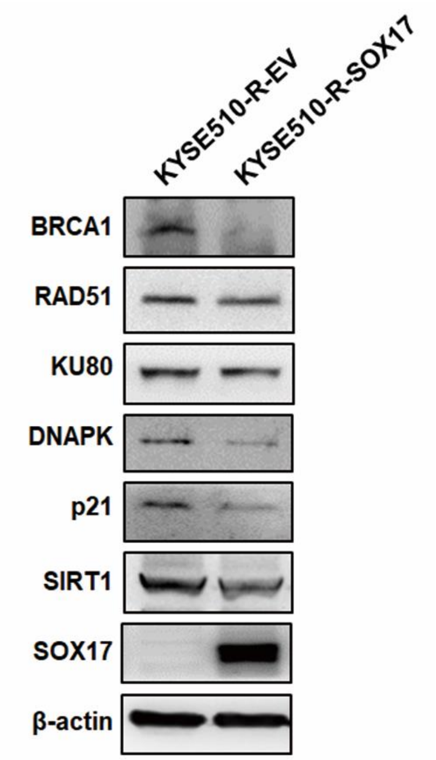

Figure S2 Overexpression of SOX17 in KYSE510-R-SOX17 cells reduced the protein expression level of DNA repair and damage-responsive genes analyzed as compared to KYSE510-R-EV cells. β-actin was used as an internal control for western blots.
